# Effect of asbestos exposure on differentiation and function of cytotoxic T lymphocytes

**DOI:** 10.1186/s12199-020-00900-6

**Published:** 2020-10-08

**Authors:** Naoko Kumagai-Takei, Yasumitsu Nishimura, Megumi Maeda, Hiroaki Hayashi, Hidenori Matsuzaki, Suni Lee, Kei Yoshitome, Tatsuo Ito, Takemi Otsuki

**Affiliations:** 1 Department of Hygiene, Kawasaki Medical School, Kurashiki, 701-0192 Japan; 2 Department of Biofunctional Chemistry, Graduate School of Environmental and Life Science, Okayama University, Okayama, 700-8530 Japan; 3 Department of Dermatology, Kawasaki Medical School, Kurashiki, 701-0192 Japan; 4 Department of Life Sciences, Faculty of Life and Environmental Sciences, Prefectural University of Hiroshima, Shobara, 727-0023 Japan

**Keywords:** Asbestos, Cytotoxic T lymphocytes, Granzyme B, Perforin, IFN-γ, Proliferation, Differentiation, Mesothelioma

## Abstract

Asbestos exposure is known to cause malignant mesothelioma, which is associated with poor prognosis. We focused on and examined the effect of asbestos exposure on the differentiation and function of cytotoxic T lymphocytes (CTLs). CTLs have the ability to specifically attack tumor cells after being differentiated from naïve CD8^+^ T cells following antigen stimulation. Exposure to chrysotile B asbestos suppressed the differentiation of CTLs during the mixed lymphocyte reaction (MLR) and was associated with a decrease in proliferation of CD8^+^ T cells. Additionally, in an effort to investigate the mechanism associated with suppressed CTL differentiation upon exposure to asbestos, we focused on IL-2, a cytokine involved in T cell proliferation. Our findings indicated that insufficient levels of IL-2 are not the main cause for the suppressed induction of CTLs by asbestos exposure, although they suggest potential improvement in the suppressed CTL function. Furthermore, the functional properties of peripheral blood CD8^+^ lymphocytes from asbestos-exposed individuals with pleural plaque (PP) and patients with malignant mesothelioma (MM) were examined. MM patients showed lower perforin levels in CD8^+^ lymphocytes following stimulation compared with PP-positive individuals. The production capacity of IFN-γ in the MM group tended to be lower compared with healthy volunteers or PP-positive individuals. In an effort to determine whether chronic and direct asbestos exposure affected the function of CD8^+^ T cells, cultured human CD8^+^ T cells were employed as an in vitro model and subjected to long-term exposure to chrysotile (CH) asbestos. This resulted in decreased levels of intracellular perforin and secreted IFN-γ. Those findings underlie the possibility that impaired CD8^+^ lymphocyte function is caused by asbestos exposure, which fail to suppress the development of MM. Our studies therefore reveal novel effects of asbestos exposure on CTLs, which might contribute towards the development and implementation of an effective strategy for the prevention and cure of malignant mesothelioma.

## Introduction

Asbestos is the commercial collective name for a group of naturally occurring fibrous silicate minerals [1, 2]. Its name comes from the Greek for “unquenchable” and the minerals are very valuable with properties such as thermal resistance, tensile strength, flexibility, adiabaticity, sound and electrical insulation, durability, and resistance to chemical attack [1]. Asbestos has been used by humans for over 6000 years and was extensively commercially mined and manufactured in earnest in the latter part of the nineteenth century [1]. A great deal of asbestos was also used in the construction trades and in the shipbuilding industry [3]. However, once health risks were identified with asbestos, the use of all forms of asbestos was banned in some countries about 35 years ago [3]. Currently, more than 50 countries including Japan have banned asbestos, with the exclusion of the USA and India, to name a few [3, 4]. Major asbestos-producing countries like Russia, Kazakhstan, China, and Brazil continue to produce and export asbestos to countries all over the world [3]. As a result, it is considered that asbestos has continued to be used in many parts of Asia, Africa, and some countries of Latin America [3]. China has also been a major consumer of asbestos like India [3]. Therefore, malignant mesothelioma associated with occupational or environmental asbestos exposure is a global problem that needs to be solved [5, 6].

As mentioned above, although asbestos possesses a variety of beneficial properties, it is believed that certain health risks such as the development of malignant mesothelioma are associated with asbestos exposure [7, 8]. Wagner et al. reported for the first time that asbestos exposure is associated with mesothelioma in 1960 [9]. Subsequent epidemiological and case-control studies have documented rising rates of malignant mesothelioma (MM) following the heavy commercial use of asbestos in many industrialized nations [7]. Gemba et al. reported that more than 70% of MM cases were associated with occupational or environmental asbestos exposure in Japan from a retrospective survey based on records for 6030 cases of death due to MM between 2003 and 2008 [6, 10]. Additionally, it is known that mesothelioma takes about 40 years from initial exposure to onset [11–14]. In fact, Murayama T. has estimated that the deaths of MM in Japan will peak around the year 2030 [5], although the use of asbestos is currently prohibited in Japan. Moreover, malignant pleural mesothelioma is a highly aggressive tumor with poor prognosis; the median overall survival is only about 12 months [6].

Many studies have focused on documenting the characteristics of asbestos fibers and delineating the mechanism associated with asbestos-mediated carcinogenesis. Asbestos fibers cause chromosomal changes, DNA damage, and oxidative DNA lesions at cytotoxic concentrations in mesothelial cells in vitro [15, 16]. However, the onset of malignant mesothelioma requires a prolonged period of time to elapse, as mentioned above [11–14]. This suggests the possibility that the development of malignant mesothelioma might be related to alterations in other functions such as biological defense mechanisms. Therefore, it occurred to us that antitumor immunity might be attenuated by exposure to inhaled asbestos. On the basis of this hypothesis, our investigations revealed alterations in the expression pattern of natural killer (NK) cell-activating receptors on human NK cells and functions in CD4^+^ T cells following exposure to asbestos [17, 18]. However, the effect of asbestos fibers on CD8^+^ CTL differentiation and function has hitherto not been examined by us or other researchers. Recently, Yamada et al. reported that the presence of a high density of CD8^+^ tumor-infiltrating lymphocytes was a significantly better prognostic factor for the survival of patients with malignant pleural mesothelioma following extrapleural pneumonectomy [19]. This supports our idea that CD8^+^ T cells play an important role in antitumor immunity against malignant pleural mesothelioma. In this paper, we introduce our findings and considerations regarding asbestos-induced alterations in CTL differentiation and function.

## Role of CTLs in antitumor immunity

In antitumor immunity, CD8^+^ T cells as well as NK cells play a role as effectors which kill tumor cells [20]. NK cells orchestrate innate immune responses and act at the front line of tumor immunity as first effectors to kill transformed cells [21], although NK cells are incapable of rearranging receptors for antigen recognition like T cells through their T cell receptors (TCRs) and are unable to recognize antigens displayed in the context of classical major histocompatibility complex (MHC) molecules [22]. On the other hand, CTLs have antigen-specific TCRs on the cell surface, which can recognize cognate peptides bound on MHC class-I molecules expressed on the target cell, thereby allowing CTLs to attack the target cells in an antigen-specific manner [23]. It is well known that CTLs are differentiated from naïve CD8^+^ T cells following antigen stimulation. When these naïve cells encounter antigen, they are immediately activated, expand, and differentiate into antigen-specific effector/memory CTLs, which gain the ability to produce effector molecules, such as perforin and granzyme B, as well as cytokines, such as interferon-gamma (IFN-γ) and tumor necrosis factor-alpha (TNF-α) [24]. Following attachment of CTLs to a target cell, they transport lytic granules to the adhesion site with the target and release the granule contents, perforin, and granzyme B into the immune synapse between the CTLs and the target. The granule contents then act on the target cell to induce apoptosis [25].

## Effect of asbestos exposure on differentiation of cytotoxic T lymphocytes

**Fig. 1:**
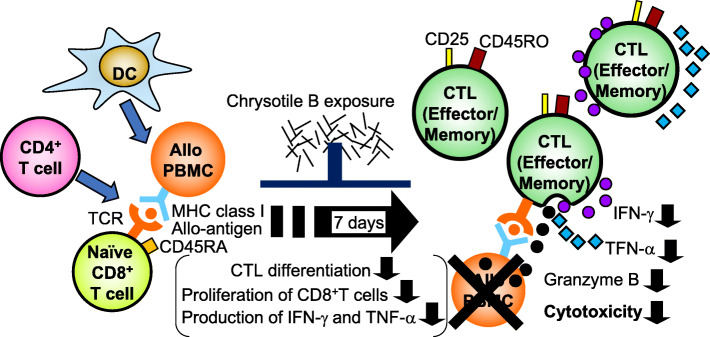
Immune-suppressive effect of asbestos exposure. The illustration shows that asbestos exposure has the potential to impair the differentiation of CTLs with suppressed proliferation of CD8^+^T cells. As demonstrated by our study, asbestos exposure results in lower cytotoxicity for allogenic targets in PBMCs exposed to CB, but not CR, compared with non-exposed PBMCs during the MLR. In particular, exposure to CB during the MLR suppressed the increase in granzyme B^+^ and IFN-γ^+^ cells. CB exposure also suppressed the increase in CD45RO^+^ effector/memory and CD25^+^-activated cells in CD8^+^ lymphocytes, and the decrease in CD45RA^+^ cells. Moreover, the production of IFN-γ and TNF-α decreased in the presence of CB

It is known that inhaled asbestos fibers translocate from the lung to the lymph node through the lymphatic system [26, 27]. The lymph node is a place where naïve CD8^+^ T cells encounter an antigen and differentiate into effector CTLs [28]. Therefore, we hypothesized that differentiation of CTLs might be impaired upon exposure to asbestos accumulated in the body. We first examined the effect of asbestos exposure on the differentiation of human CTLs. A mixed lymphocyte reaction (MLR) with peripheral blood mononuclear cells (PBMCs) was performed to efficiently and conveniently induce CTL differentiation [29, 30]. In the MLR, irradiation-induced allogenic PBMCs stimulate naïve CD8^+^ T cells in PBMCs to be differentiated into allo-antigen-specific CTLs with cell proliferation. In an effort to investigate the effect of asbestos exposure on CTL differentiation, PBMCs were cultured and stimulated with irradiated allogenic PBMCs for 7 days in the absence or presence of chrysotile B (CB) or crocidolite (CR) asbestos at 5 μg/ml. As a negative control for CTL differentiation, allogenic PBMCs were not included in the culture. It was found that the number of CD3^+^CD8^+^ cells was higher in PBMCs stimulated with allogenic PBMCs compared with those in the absence of allogenic cells. PBMCs exposed to CB asbestos during the MLR exhibited a significant decrease in the number of those cells. We evaluated the cytotoxicity against allogenic target cells by flow cytometry (FCM) and measured the number of fluorescently-labeled target cells that were attacked and killed. PBMCs harvested after the MLR exhibited a significant level of allogenic cytotoxicity. In contrast, PBMCs exposed to CB asbestos during the MLR exhibited markedly decreased cytotoxicity. However, exposure to CR asbestos did not cause a decrease in cytotoxicity as observed with CB exposure. Then, to remove the quantitative difference in CD8^+^ T cells between samples of harvested PBMCs and to examine cytotoxic performance per cell, we purified CD8^+^ lymphocytes from cultured PBMCs and prepared cell suspensions of CD8^+^ lymphocytes as effectors for killing assays against allogenic targets. CD8^+^ cells exposed to CB asbestos during the MLR showed lower cytotoxicity compared with CD8^+^ cells following the MLR without CB. These results indicate that exposure to CB asbestos causes a decreased induction of allogenic cytotoxicity exerted by CD8^+^ T cells. Allogenic stimulation using the MLR induced increases in the percentage of granzyme B and IFN-γ in CD8^+^ lymphocytes. However, exposure to CB asbestos during the MLR significantly suppressed these increases in CD8^+^ lymphocytes. Allogenic stimulation also induced a decrease in naïve CD45RA^+^ cell levels and an increase in effector/memory CD45RO^+^ and activated CD25^+^ cells, which means that allogenic stimulation induced activation of naïve CD8^+^ T cells and their differentiation into effector/memory cells. In contrast, it was found that exposure to CB during the MLR suppressed differentiation into CTLs. Additionally, we found that CB exposure suppressed the proliferation of CD8^+^ T cells without an increase in apoptosis, which was associated with suppressed differentiation of CTLs upon exposure to CB. It is known that IFN-γ, TNF-α, and IL-2 cytokine production are required for the promotion of CTL differentiation and the proliferation of CD8^+^T cells [31, 32]. Therefore, the supernatants from cultures of PBMCs following allogenic stimulation were harvested and examined for the production of those cytokines. The production of IFN-γ and TNF-α, but not of IL-2, decreased in the presence of CB asbestos. Taken together, these results indicate that exposure to asbestos has the potential to impair the differentiation of effector cytotoxic T lymphocytes and is accompanied by decreases in cell proliferation as well as the production of IFN-γ and TNF-α, as shown in Fig. 1 [33].

## IL-2 addition to the suppressed differentiation of CTLs upon exposure to asbestos

IL-2 is a cytokine required for CD8^+^T cells to proliferate during development into CTLs [34]. Therefore, in an effort to investigate the mechanism of asbestos-induced suppressed differentiation of CTLs with decreased proliferation of CD8^+^ T cells, we examined whether IL-2 insufficiency might contribute to the suppressed induction of CTLs upon exposure to asbestos. When IL-2 was added at 1 ng/mL on the second day of the MLRs upon exposure to CB asbestos, IL-2 addition did not result in the recovery of asbestos-induced decreases in the proliferation and percentage of CD25^+^ and CD45RO^+^ cells in CD8^+^ lymphocytes. On the other hand, the decrease in the percentage of granzyme B^+^ cells was partially recovered and the cytotoxicity was improved by the addition of IL-2. These findings indicate that IL-2 insufficiency is not the main cause for the suppressed differentiation of CTLs upon exposure to asbestos, although suppressed CTL function might be recoverable, as summarized in Table 1 [35].

**Table 1:** IL-2 addition to cultures upon exposure to chrysotile B

Parameters	Recover by IL-2 addition?
Cell number of CD3^+^CD8^+^ cells	No
% CD45RA^+^ cells	No
% CD45RO^+^ cells	No
% CD25^+^ cells	No
Proliferation	No
% Granzyme B^+^ cells	Yes, partially
Cytotoxicity	Yes
**Parameters**	**Induction by IL-2 addition?**
%Granzyme B^+^ cells in non-proliferating CD8^+^ cells	Yes
%Granzyme B^+^ cells in proliferating CD8^+^ cells	No

## Characteristics and function of CD8^+^ lymphocytes in PP individuals and MM patients

Based on the finding that CTL differentiation is suppressed upon exposure to asbestos, we speculated that the functional properties of CD8^+^ lymphocytes might be diminished in asbestos-exposed individuals with pleural plaque (PP) or patients with MM. The presence of PP indicates previous asbestos inhalation and is known to be whitish, sharply circumscribed, fibrous, hyaline, and sometimes calcified and form patches involving parietal pleura, although one report has indicated that pleural plaques are harmless in and of themselves [36]. Therefore, we examined the characteristics and function of CD8^+^ lymphocytes in PBMCs using FCM, and compared them among PP individuals, MM patients, and healthy volunteers (HV). Some of the PBMCs were stimulated with phorbol 12-myristate 13-acetate (PMA)/ionomycin as a substitute for antigenic stimulation. The percentage of CD45RA^−^ cells was examined as memory cells. All of the PP individuals who provided blood specimens did not suffer from any tumors. The number of CD3^+^CD8^+^ cells per 1 mL of blood in the PP and MM groups was significantly lower than that of HV. Degranulation in stimulated CD8^+^ lymphocytes, which is identified by cell surface expression of CD107a following stimulation, did not differ among the three groups. The production of IFN-γ in the MM group tended to be lower than that of the HV and PP groups, although there was no statistically significant difference among the three groups. In contrast, the MM group showed significantly lower levels of perforin in CD8^+^ lymphocytes after stimulation compared with PP individuals. These results indicate that CD8^+^ lymphocytes in MM patients possess a lower ability to retain and enhance intracellular perforin levels following stimulation compared with PP patients and HV, which indicates impairment of peripheral blood CD8^+^ lymphocyte cytotoxicity in MM patients [37]. Additionally, the following characteristic of CD8^+^ lymphocytes was common between PP-positive individuals and MM patients. The percentage of perforin^+^ cells and CD45RA^−^ cells in circulating CD8^+^ lymphocytes of the PP and MM groups was higher compared with that of HV. These findings are summarized in Table 2. The differences between the PP and MM groups underlie the immunological state related to the pathology of malignant mesothelioma.

**Table 2:** Functional properties of CD8^+^ lymphocytes in individuals with pleural plaque and patients with malignant mesothelioma

Parameters		HV	PP	MM
CD3^+^CD8^+^ (%)		+	+	+
CD107a^+^ (%)		+	+	+
IFN-γ^+^ (%)		+	+	+
Granzyme B^+^ (%)	Before	+	+	+
	After	+	++	+
	After-Before	+	++	+
Perforin^+^ (%)	Before	++	+++	+++
	After	++	+++	++
	After-Before	++	++	+
CD45RA^-^ (%)		+	++	++
CD3^+^CD8^+^ (cell number)		++	+	+

*HV* healthy volunteers, *PP* asbestos-exposed individuals with pleural plaque, *MM* asbestos-exposed patients with malignant mesothelioma, *Before* the percentage of granzyme B^+^ cells in fresh CD8^+^ lymphocytes, *After* the percentage of granzyme B^+^ cells in stimulated CD8^+^ lymphocytes, *After-Before* the percentage of granzyme B^+^ or perforin^+^ cells in fresh CD8^+^ lymphocytes was subtracted from the percentage of granzyme B^+^ or perforin^+^ cells in stimulated cells

## Relationship between suppressed CD8^+^ lymphocyte function and asbestos exposure in MM patients

While the MM group showed impaired CD8^+^ lymphocyte function after stimulation, we questioned whether this reduced function might be related to immunosuppression by tumor cells [38] and not be caused by asbestos exposure. In fact, a study using PBMCs from patients with carcinoma showed reduced T cell receptor-mediated cytotoxicity [39]. Therefore, we investigated whether chronic and direct asbestos exposure affected the function of CD8^+^ T cells. For this purpose, an in vitro model was prepared by culturing the human CD8^+^ T cell line EBT-8 for 1 to 2 months with long-term exposure to chrysotile (CH) asbestos. As a control, the cell subline cultured in the absence of asbestos was also performed. Cultured cells were assayed for intracellular levels of perforin, granzyme B, and IFN-γ and for degranulation and production of INF-γ. Exposure to CH asbestos at 5 μg/ml or 30 μg/ml had no effect on intracellular granzyme B levels. In contrast, exposure to CH asbestos at both concentrations resulted in decreased levels of perforin, whereas degranulation following stimulation with antibodies to CD3 remained unaffected, even with exposure to CH asbestos at a concentration of 30 μg/ml. CH exposure at 30 μg/ml also decreased levels of secreted IFN-γ stimulated via CD3, although there was no difference in the percentage of IFN-γ^+^ cells induced by PMA/ionomycin (Table 3) [40]. These results indicate that perforin levels and the production of IFN-γ in human CD8^+^ T cells could be potentially suppressed by long-term exposure to asbestos. It is obvious that lymphocytes encounter asbestos prior to tumor cell formation in the body. Therefore, it is possible that asbestos exposure causes impaired CD8^+^ lymphocyte function that results in failure to suppress the development of MM, although it is possible that the characteristics of CD8^+^ T cells in MM patients might be affected by immunosuppression derived from the tumor.

**Table 3:** Functional properties of human CD8^+^ T cell line following long-term exposure to asbestos fibers

Parameters	EBT-8-Org	EBT-8-CH5 or/and EBT-8-CH30	Significance
Granzyme B (MFI)	++ or +	+ or ++	N.S.
Perforin^+^ (%)	++	+	*p* < 0.01
IFN-γ^+^ (%)	++ or +	+ or ++	N.S.
Production of IFN-γ	++	+	*p* < 0.0001
Degranulation (%)	+	+	N.S.

*MFI* mean fluorescence intensity, *N.S.* not significant

The human CD8+ T cell line was then cultured in the absence or presence of CH asbestos at low or middle concentrations of 5 or 30 μg/ml for 1 to 2 months, with cell groups being referred to as EBT-8-Org, EBT-8-CH5, and EBT-8-CH30, respectively

## Conclusion

Our present investigation is the first to demonstrate that exposure to asbestos has the potential to suppress the differentiation and function of human CTLs using in vitro human cell culture models and CD8^+^lymphocytes derived from peripheral blood of individuals with pleural plaque and patients with malignant mesothelioma. Taken together, our findings can help asbestos-exposed individuals when employing immunological markers for the early diagnosis of malignant mesothelioma as peripheral blood is relatively easy to collect. These findings might also contribute to an evaluation of the efficacy of strategies involving the immunotherapy of malignant mesothelioma in reverse. Future studies will continue with investigations attempting to delineate the mechanism of suppressed differentiation and function of CTLs upon exposure to asbestos.

## Data Availability

Not applicable.

## Abbreviations

CTL: Cytotoxic T lymphocytes
MLR: Mixed lymphocytes reaction
PP: Pleural plaque
MM: Malignant mesothelioma
CH: Chrysotile
NK: Natural killer
TCRs: T cell receptors
MHC: Major histocompatibility complex
IFN-γ: Interferon-gamma
TNF-α: Tumor necrosis factor-alpha
PBMC: Peripheral blood mononuclear cells
CB: Chrysotile B
CR: Crocidolite
FCM: Flow cytometry
IL-2: Interleukin-2
HV: Healthy volunteers
PMA: Phorbol 12-myristate 13-acetate

## References

[1] King JE, Hasleton PS, O’Byrne K, Rusch V (2006). The epidemiology and aetiology of malignant mesothelioma. Malignant pleural mesothelioma.

[2] Kumagai-Takei N, Maeda M, Chen Y, Matsuzaki H, Lee S, Nishimura Y, et al. Asbestos induces reduction of tumor immunity. Clin Dev Immunol. 2011. 10.1155/2011/481439. 10.1155/2011/481439 PMC3189469 22007251

[3] Frank AL, Joshi TK (2014). The global spread of asbestos. Ann Glob Health.

[4] Furuya S, Takahashi K. Experience of Japan in achieving a total ban on asbestos. Int J Environ Res Public Health. 2017. 10.3390/ijerph14101261. 10.3390/ijerph14101261 PMC5664762 29053631

[5] Nishimura Y, Kumagai-Takei N, Lee S, Yoshitome K, Otsuki T, Otsuki T (2020). Suppressed immune system caused by exposure to asbestos and malignant mesothelioma. Asbestos-related diseases.

[6] Fujimoto N, Otsuki T (2020). Immunocheckpoint blockade in malignant mesothelioma. Asbestos-related diseases.

[7] Sporn TA, Roggli VL. Mesothelioma. In: Roggli VL, Oury TD, and Sporn TA, editors. Asbestos-associated diseases. New York: Springer-Verlag. 2004. p104-168.

[8] Mossman BT, Kamp DW, Weitzman SA (1996). Mechanisms of carcinogenesis and clinical features of asbestos-associated cancers. Cancer Investig.

[9] Wanger JC, Sleggs CA, Marchand P (1960). Diffuse pleural mesothelioma and asbestos exposure in the North Western Cape Province. Br J Ind Med.

[10] Gemba K, Fujimoto N, Kato K, Aoe K, Takeshima Y, Inai K (2012). National survey of malignant mesothelioma and asbestos exposure in Japan. Cancer Sci.

[11] McDonald AD, McDonald JC (1978). Mesothelioma after crocidolite exposure during gas mask manufacture. Environ Res.

[12] Selikoff IJ, Hammond EC, Seidman H (1979). Mortality experience of insulation workers in the United States and Canada, 1943--1976. Ann N Y Acad Sci.

[13] Selikoff IJ, Hammond EC, Seidman H (1980). Latency of asbestos disease among insulation workers in the United States and Canada. Cancer..

[14] Reid A, de Klerk NH, Magnani C, Ferrante D, Berry G, Musk AW (2014). Mesothelioma risk after 40 years since first exposure to asbestos: a pooled analysis. Thorax..

[15] Dusinská M, Collins A, Kazimírová A, Barancoková M, Harrington V, Volkovová K (2004). Genotoxic effects of asbestos in humans. Mutat Res.

[16] Topinka J, Loli P, Georgiadis P, Dusinská M, Hurbánková M, Kováciková Z (2004). Mutagenesis by asbestos in the lung of lambda-lacI transgenic rats. Mutat Res.

[17] Nishimura Y, Miura Y, Maeda M, Kumagai N, Murakami S, Hayashi H (2009). Impairment in cytotoxicity and expression of NK cell- activating receptors on human NK cells following exposure to asbestos fibers. Int J Immunopathol Pharmacol.

[18] Maeda M, Nishimura Y, Hayashi H, Kumagai N, Chen Y, Murakami S (2011). Reduction of CXC chemokine receptor 3 in an in vitro model of continuous exposure to asbestos in a human T-cell line, MT-2. Am J Respir Cell Mol Biol.

[19] Yamada N, Oizumi S, Kikuchi E, Shinagawa N, Konishi-Sakakibara J, Ishimine A (2010). CD8 (+) tumor-infiltrating lymphocytes predict favorable prognosis in malignant pleural mesothelioma after resection. Cancer Immunol Immunother.

[20] Banchereau J, Palucka AK (2005). Dendritic cells as therapeutic vaccines against cancer. Nat Rev Immunol..

[21] Nishimura Y, Maeda M, Kumagai-Takei N, Lee S, Matsuzaki H, Wada Y, et al. Altered functions of alveolar macrophages and NK cells involved in asbestos-related diseases. Environ Health Prev Med. 2013; 18:198-204, doi 10.1007/s12199-013-0333-y. 10.1007/s12199-013-0333-y PMC3650181 23463177

[22] Hallett WHD, Murphy WJ (2004). Natural killer cells: biology and clinical use in cancer therapy. Cell Mol Immunol.

[23] Uzhachenko RV, Shanker A. CD8^+^ T lymphocyte and NK cell network: circuitry in the cytotoxic domain of immunity. Front Immunol. 2019. 10.3389/fimmu.2019.01906. 10.3389/fimmu.2019.01906 PMC6700470 31456803

[24] Harty JT, Tvinnereim AR, White DW (2000). CD8^+^ T cell effector mechanisms in resistance to infection. Annu Rev Immunol.

[25] Catalfamo M, Henkart PA (2003). Perforin and the granule exocytosis cytotoxicity pathway. Cur Opin Immunol.

[26] Dodson RF, Williams MG, Corn CJ, Brollo A, Bianchi C (1991). A comparison of asbestos burden in lung parenchyma, lymph nodes, and plaques. Ann N Y Acad Sci.

[27] Miserocchi G, Sancini G, Mantegazza F, Chiappino G (2008). Translocation pathways for inhaled asbestos fibers. Environ Health.

[28] Heath WR, Carbone FR (2001). Cross-presentation in viral immunity and self-tolerance. Nat Rev Immunol..

[29] Rentenaar RJ, Vosters JL, van Diepen FN, Remmerswaal EB, van Lier RA, ten Berge IJ (2002). Differentiation of human alloreactive CD8(+) T cells in vitro. Immunology..

[30] Melief CJ, de Waal LP, van der Meulen MY, Melvold RW, Kohn HI (1980). Fine specificity of alloimmune cytotoxic T lymphocytes directed against H-2K: a study with kb mutants. J Exp Med.

[31] Cox MA, Harrington LE, Zajac AJ (2011). Cytokines and the inception of CD8 T cell responses. Trends Immunol.

[32] Brossart P, Bevan MJ (1997). Presentation of exogenous protein antigens on major histocompatibility complex class I molecules by dendritic cells: pathway of presentation and regulation by cytokines. Blood..

[33] Kumagai-Takei N, Nishimura Y, Maeda M, Hayashi H, Matsuzaki H, Lee S (2013). Effect of asbestos exposure on differentiation of cytotoxic T lymphocytes in mixed lymphocyte reaction of human peripheral blood mononuclear cells. Am J Respir Cell Mol Biol.

[34] Lai Y-P, Lin C-C, Liao W-J, Tang C-Y, Chen S-C. CD4+ T cell-derived IL-2 signals during early priming advances primary CD8+ T cell responses. PLoS One. 2009;4. 10.1371/journal.pone.0007766. 10.1371/journal.pone.0007766 PMC2770320 19901991

[35] Kumagai-Takei N, Nishimura Y, Matsuzaki H, Lee S, Yoshitome K, Hayashi H, et al. The suppressed induction of human mature cytotoxic T lymphocytes caused by asbestos is not due to interleukin-2 insufficiency. J Immunol Res. 2016; doi.org/10.1155/2016/7484872. 10.1155/2016/7484872 PMC5126420 27975069

[36] Hillerdal G, Henderson DW (1997). Asbestos, asbestosis, pleural plaques and lung cancer. Scand J Work Environ Health.

[37] Kumagai-Takei N, Nishimura Y, Maeda M, Hayashi H, Matsuzaki H, Lee S, et al. J Immunol Res. 2014. doi.org/10.1155/2014/670140. 10.1155/2014/670140 PMC4087265 25045719

[38] Dunn GP, Koebel CM, Schreiber RD (2006). Interferons, immunity and cancer immunoediting. Nat Rev Immunol.

[39] Crocenzi TS, Tretter CPG, Schwaab T, Schned AR, Heaney JA, Cole BF (2005). Impaired cytolytic activity in peripheral blood T cells from renal cell carcinoma patients. Clin Immunol.

[40] Kumagai-Takei N, Nishimura Y, Matsuzaki H, Lee S, Yoshitome K, Otsuki T. Decrease in intracellular perforin levels and IFN-γ production in human CD8+ T cell line following long-term exposure to asbestos fibers. J Immunol Res. 2018; doi.org/10.1155/2018/4391731. 10.1155/2018/4391731 PMC6218727 30426024

